# Effects of Combined Endurance and Resistance Training in Women With Multiple Sclerosis: A Randomized Controlled Study

**DOI:** 10.3389/fneur.2021.698460

**Published:** 2021-08-05

**Authors:** Luca Correale, Cosme Franklim Buzzachera, Giulia Liberali, Erwan Codrons, Giulia Mallucci, Matteo Vandoni, Cristina Montomoli, Roberto Bergamaschi

**Affiliations:** ^1^Department of Public Health, Experimental and Forensic Medicine, University of Pavia, Pavia, Italy; ^2^Inter-Department Multiple Sclerosis Research Centre, National Neurological Institute Casimiro Mondino, Pavia, Italy; ^3^Laboratory of Adapted Motor Activity, Department of Public Health, Experimental & Forensic Medicine, University of Pavia, Pavia, Italy

**Keywords:** multiple sclerosis, strength training, aerobic training, training adaptation, muscle strength

## Abstract

**Purpose:** To test the hypothesis that combined resistance and endurance training would improve muscle strength, fatigue, depression, and quality of life in persons with MS.

**Methods:** Twenty-seven women with MS were randomly assigned to either control (CON, *n* = 13) or the experimental (EXP, *n* = 14) group. The participants in the EXP group trained twice a week for 12 weeks, followed by 12 weeks of detraining. Both CON and EXP groups were tested before and after 12 weeks of the intervention period, as well as 12 weeks after training cessation (follow-up), where measures of muscle strength, fatigue, depression, and quality of life were evaluated.

**Results:** There were significant changes in maximal voluntary isometric contraction (MVIC), 1RM leg extension, and 1RM chest press following the intervention period in the EXP group (*P* < 0.05), but not in the CON group (*P* > 0.05). These changes persisted after 12 weeks of detraining. Similar findings were found for fatigue, depression, and physical and mental composites of quality of life.

**Conclusion:** These results suggest that combined exercise training, at a minimum, prevents the disease-related deterioration of muscular performance and quality of life and well-being in persons with MS.

## Introduction

Multiple sclerosis (ms) is described as a chronic autoimmune, inflammatory neurologic disease of the central nervous system. In most persons with ms, the onset of the disease is characterized by sporadic episodes of reversible neurological deficits, followed by progressive neurological deterioration over time (1). ms is currently considered the most common inflammatory neurological disease in young adults and typically presents in persons 20 to 45 years of age, with occasional cases in childhood or late middle age, potentially causing severe neurological disability throughout adult life. Of note, like many other autoimmune diseases, women are affected two to three times more often than men (2). Data from the Global Burden of Diseases study estimates that in 2016, ms may have affected 2.2 million people worldwide, mainly from North America and European countries. ms also contributed 0.04% of total disability-adjusted life year and 0.05% of all lost years of life in 2016 (3).

The clinical manifestations of ms have been well documented and include sensory, motor, cerebellar, or visual system abnormalities. The occurrence of ms-related manifestations varies between individuals, based on the extent and location of the lesions and disease exacerbations and progression (4). Most persons with ms may experience, for example, reduced muscle strength during dynamic and isometric muscle contractions (5). The reasons for the strength impairments in this population are complex and still not completely understood. Previous research, however, has shown that mechanisms of both neural and muscular origin appear to be involved (6). Such mechanisms include decreased motor unit firing rates (7), abnormal motor unit recruitment patterns (7), prolonged central motor conduction time (8), slowing of muscle contractile properties (9), impaired muscle oxidative capacity (9), among others. There is mounting evidence that strength impairments in people with ms are also related to the loss of muscle mass per se (10). Whether these ms-related changes in terms of structure and function of skeletal muscle occur due to the disease, muscle disuse, or both are uncertain. It is recognized, however, that detrimental changes in muscle mass and muscle strength impact negatively on the quality of life and overall well-being of persons with ms.

Both pharmacologic and non-pharmacologic approaches are used to manage symptoms experienced by persons with ms, with the scope of maintaining or improving function while preserving the quality of life. Pharmacologic interventions are likely to control inflammatory activity but not neurodegenerative processes, as there is no cure for the disease (11). Hence, most people with ms still experience residual symptoms and dysfunctions. Non-pharmacologic interventions, otherwise, might be useful as behavioral approaches for managing clinical manifestations of the disease, with minimal adverse side-effects. Some non-pharmacologic approaches, such as resistance training, can be beneficial in maintaining muscle mass and muscle strength in persons with ms (12–14). Resistance training has also proven to be effective in reducing muscle weakness (6), improving balance (15), and decreasing perceived fatigue (12, 14), with positive impacts on activities of daily living, quality of life, and well-being of people with ms (12, 13). Resistance training is, therefore, an effective and well-tolerated non-pharmacologic intervention in this population, with exciting possibilities in the management of ms-related manifestations.

Endurance training is another appropriate non-pharmacologic intervention for persons with ms. There is mounting evidence that endurance training induces improvements in aerobic fitness (16, 17) and measures of health-related quality of life (17, 18), with discrete effects on functional capacity (17). The reported effects of endurance training on muscular performance (18, 19) and perceived fatigue (16) are also modest. However, it has been speculated that endurance training, combined with resistance training, may further augment the adaptations to exercise training in persons with ms (20). Unfortunately, to the best of our knowledge, there are currently few published reports (19, 21, 22) on the effects of combined exercise training in this population, making robust conclusions on the issue difficult without further research. The purpose of this study was, therefore, to investigate the effects of the combined endurance and resistance training on muscle strength, perceived fatigue, depression, and quality of life in women with ms. The residual effects of the combined exercise training were also investigated following a period of training cessation. We hypothesized that, compared with the control condition, combined endurance and resistance training would promote gains in muscle strength (12–14, 18, 19) and improvements in quality of life (12, 17, 18), in combination with attenuated symptoms of both perceived fatigue (14) and depression (23). We also hypothesized that a subsequent detraining period would reduce, but not mitigate, most of the benefits caused by the combined exercise training (12).

## Materials and Methods

### Experimental Design

**Figure 1:**
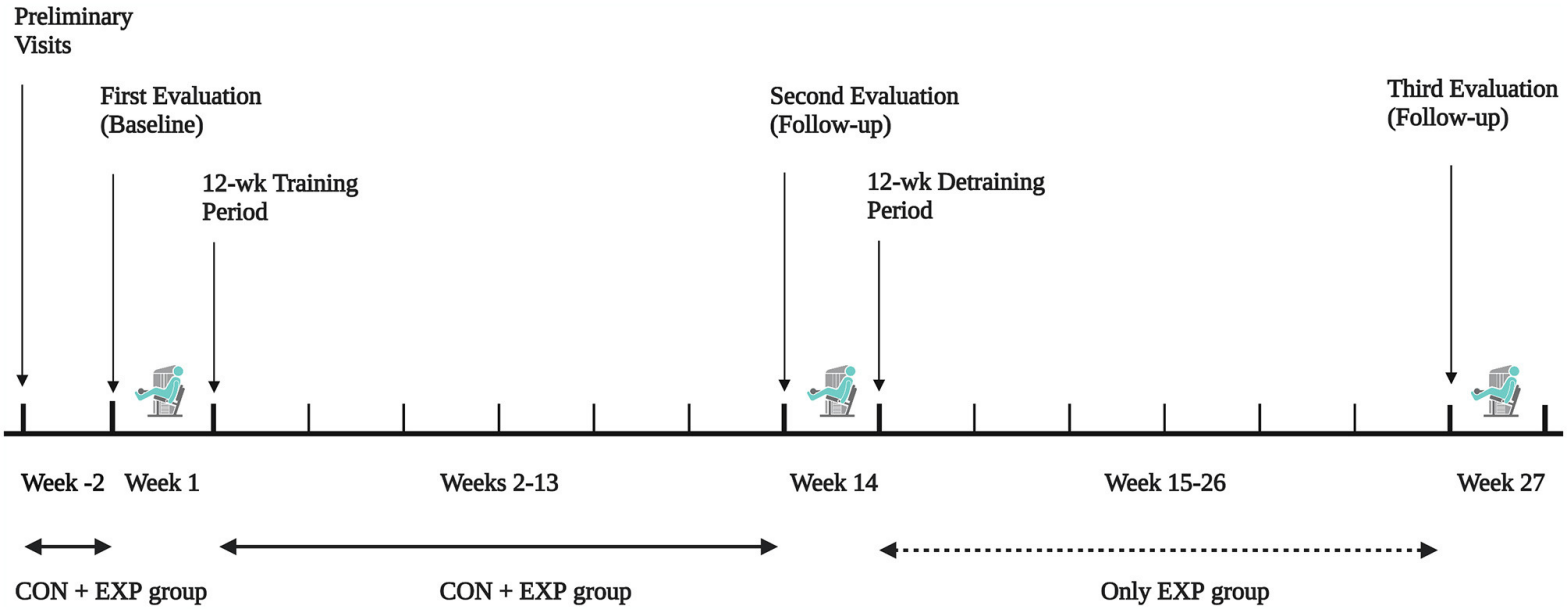
Schematic overview of study timeline.

This study used a randomized controlled trial design to test the effectiveness of a combined endurance and resistance training to improve muscle strength, perceived fatigue, depression, and quality of life in women with ms. Before and after training, participants were tested using identical protocols (see Measurements section for details). Baseline testing was completed during the first week of the study, during which time no endurance or resistance training was carried out. This was followed by a 12-wk period of supervised, combined endurance and resistance training, with the testing procedures repeated after the last week of training. The residual effects of the combined exercise training were further investigated following a 12-wk period of training cessation, with the testing procedures repeated after the last week of detraining (see Figure 1 for details). The total duration of the present study was, therefore, 27 weeks, consisting of a 12-wk combined exercise training period followed by a 12-wk detraining period, with the measurements repeated at 12-wk intervals (i.e., weeks 1, 14, and 27).

### Ethical Approval

The current research was reviewed by the reference Ethical committee (protocol number 20170002431) and conducted following the latest revision of the Declaration of Helsinki. All volunteers gave their written, informed consent before the commencement of the study, once the experimental procedures, associated risks, and benefits of participation had been explained in detail.

### Participants

A group of women were recruited to participate in this study. To be eligible, they needed to fulfill the following criteria: definite relapsing-remitting ms according to 2010 McDonald's criteria (24); Expanded Disability Status Scale score <4, with pyramidal function between 1 to 3 (25); independent ambulation without uses of unilateral assistance; age >18 and <60 years; and acceptance of treatment (see Supplementary Table for details). Potential participants were also excluded if they had neuropathic pain of the lower limbs, severe cognitive impairments, alcoholism, medical comorbidities and/or a medical condition contraindicating participation in the study, had experienced an ms attack within the past eight weeks, or were pregnant. They were also excluded if they have engaged in regular exercise over the past six months. All participants were recruited from those referred to the neurologist of the IRCCS Casimiro Mondino Foundation of Pavia for periodic clinical and electrophysiological evaluations.

### Procedures

Before starting the study, participants reported to the laboratory on two separate occasions over a 2-wk period. During the first visit, they underwent screening and anthropometric measurements. Height (cm) and weight (kg) were measured using a portable stadiometer and a calibrated scale, respectively. Body mass index (bmi; kg.m^−2^) was calculated as weight divided by height squared. Fat mass and fat-free mass were estimated (kg) using a standard tetrapolar technique (bia101, Akern, Firenze, Italy). During the second visit, participants were familiarized with the experimental procedures. They were also instructed to refrain from exercise and to avoid alcoholic and caffeinated products 24 h preceding the testing procedures. The preliminary visits were scheduled on different days with at least 48–96 h in-between.

After baseline testing, participants were ranked on one-repetition maximum (1rm) action on the leg extension, and then matched pairs were randomly allocated to either the experimental (exp) or the control (con) group. A trained research staff member, blinded to this group allocation, conducted the baseline and follow-up testing procedures. The participants in the exp group were asked to report to the training facility on nonconsecutive days, two times per week for 12 wk, to perform a combination of endurance and resistance training, lasting from 45 to 60 minutes. Each training session began with a 5-min warm-up, where participants completed a moderate-intensity aerobic exercise (~50% heart rate reserve, hrr) on either a motorized treadmill or a cycle ergometer. All participants were then asked to complete a 25-min aerobic training at a moderate-to-vigorous exercise intensity (50–70%hrr), with the heart rate being monitored continuously throughout each session. The exercise intensity was progressively increased or decreased every 2-wk based on heart rate responses (26). The endurance training was followed by resistance training, consisting of calisthenics, dumbbells, and elastic band exercises for the major muscle groups, with participants being instructed to complete three sets of 8–12 repetitions for each exercise. The rest period between sets and exercises was 60–90 s. The load was increased when three sets of 12 repetitions of an exercise could be easily completed (26). All training sessions were conducted at the same time of the day under similar environmental conditions and supervised by a trained research staff member. All participants had to attend a minimum of 90% of the scheduled training sessions to be considered compliant. During both training and detraining periods, participants were instructed to maintain their normal daily activities and dietary patterns.

### Measurements

Maximal voluntary isometric contraction (mvic) is a common method of measurement of muscle strength in persons with ms (27). A calibrated load cell (MuscleLab, Ergotest technology, Norway) recorded muscle force (N) during an isometric voluntary contraction of the knee extensors in a leg extension machine (Technogym, Gambottola, Italy). All participants were instructed to sit with hips and knees flexed 90°, with the axis of the lever arm of the leg extension machine visually aligned with the center of rotation of the knee joint. The tibial pad was adjusted proximal to the medial malleolus, and the average distance between the axis of rotation and the tibial pad was recorded for each participant. During contractions, all participants were encouraged to maintain maximal strength against the fixed lever arm of the machine for 5 s. They performed two attempts interspersed with 3 min recovery, and the highest value of muscle force was considered for analysis. Torque (N.m^−1^) was calculated as the product of the highest value of muscle force and the distance between the axis of rotation of the knee joint and the tibial pad (27).

Maximal dynamic strength was assessed using 1rm actions on the bilateral leg extension, chest press, and seated row exercise machines (Technogym, Gambottola, Italy). In brief, 1rm is currently defined as the heaviest load that can be lifted only once through a full range of motion. Additionally, 1rm testing procedures are recommended by the American College of Sports and Medicine (26) and have been previously used in ms-related studies (12, 28). Before each 1rm test, participants were allowed to perform a standard warm-up, consisting of five repetitions at loads varying from 40 to 60% of the perceived maximum. Four to five separate, single attempts interspersed with 3 min recovery in-between were then performed, with the load increasing after each successful performance. The last acceptable attempt with the highest possible load was defined as 1rm. Standardized instructions and strong verbal encouragement were given throughout the 1rm testing procedures.

The Italian version of the Modified Fatigue Impact Scale (mfis) (29) was used to measure perceived fatigue. This 21-item self-reported questionnaire is commonly used in clinical and research practice and assess how ms-related fatigue affects everyday life (30). Participants were asked to rate how often fatigue has affected them during the past four weeks using a 5-point Likert scoring system varying from 0 (*never*) to 4 (*almost always*). The total mfis score, therefore, can range from 0 to 84, with higher scores indicating a greater impact of ms-related fatigue on their daily life. Besides a total mfis score, scores for the physical (9 items), mental (10 items), and psychosocial (2 items) dimensions of fatigue can also be determined. In the present study, however, emphasis was placed on total mfis score because a complete description of fatigue is essential to examine the relationship between exercise training and ms-related manifestations. Participants with a total mfis score above 36 were categorized as being fatigued (31).

The presence and severity of depressive symptoms were assessed using the Italian version of the Beck Depression Inventory-II (bdi-ii) (32). This 21-item self-reported questionnaire is commonly used in clinical and research practice and describes somatic and cognitive-affective symptoms (33). Participants were asked to rate how they felt during the past two weeks using a 4-point Likert scoring system varying from 0 (*symptom absent*) to 3 (*severe symptoms*). The total bdi-ii score, therefore, can range from 0 to 63, with higher scores indicating greater symptom severity. A total bdi-ii score of 19 or above is indicative of depressive symptoms (33).

The Italian version of the Multiple Sclerosis Quality of Life Questionnaire (msqol-54) (34) was used to measure health-related quality of life. This self-reported questionnaire consists of 54 items divided into 12 multiple-item subscales and two single-item scales (35). Scores for each subscale can be weighed and summed to generate physical and mental health composite scores. Scores for physical and mental health composites can range from 0 to 100 and with higher scores indicating a better health-related quality of life (17). There is no single total msqol-54 score, but the criteria of minimum clinically significant differences of 1.5 and 2.5 points, respectively for the physical and mental health composites, was adopted (36).

### Statistical Analyses

Data are reported as mean ± sd unless otherwise stated. The normality of all data was checked by the Shapiro-Wilk test. Two-tailed, paired *t*-tests were used to determine whether the post- minus preintervention change score within each group was significant. Analysis of covariance (ancova) using baseline values as covariates was used to determine if there were significant between-group differences. This method is recommended for controlling for baseline differences in randomized controlled trials (37). When the ancova was significant, Fisher's post hoc analysis was used to determine differences between groups. 95% confidence intervals (ci) were calculated from postintervention means and sd's. Two-tailed, paired *t*-tests were also used to determine whether the post- minus predetraining change score within the exp group was significant. *P* < 0.05 was set as the criterion for statistical significance. Test-retest reliability for all studied variables was examined for the preintervention vs. postintervention measurements from the con group, using the 2k model of Weir (38) for mean differences (systematic error; repeated-measures anova) and intraclass correlation coefficients (icc's). Statistical analyses were performed using a commercially available software package (spss for Windows version 24.0, ibm®, Chicago, USA).

The sample size was estimated using G Power software, version 03.1.9.2, based on a previous study by Dalgas et al. (15) that reported a significant improvement in mvic following resistance training in persons with ms. It was estimated that a sample size of 12 per group was required to achieve a statistical power of 80% at an alpha level of 0.05, with a moderate Cohen's effect size of 0.49 (15).

## Results

A total of 27 women with ms were eligible to participate in this study, being randomized to either the con group (*n* = 13) or the exp group (*n* = 14). There were, however, dropouts due to episodes of sensory disturbances (*n* = 1) and circumstances unrelated to the study (*n* = 3). An overall attendance rate of ~94% was reported, with no exclusion of participants due to poor compliance.

Table 1 shows descriptive data of the participants in the con and exp groups. There were no significant differences in participant characteristics between the groups (*P* > 0.05).

**Table 1:** Descriptive characteristics of the participants at baseline.

**Variable**	**CON group (*n* = 9)**	**EXP group (*n* = 14)**
Age (yr)	48.3 ± 6.1	45.4 ± 7.2
Height (cm)	161.4 ± 7.0	161.7 ± 7.6
Weight (kg)	61.1 ± 13.1	57.8 ± 12.7
Body mass index (kg.m^−2^)	23.3 ± 4.1	21.9 ± 4.0
Fat mass (kg)	19.2 ± 9.6	15.4 ± 8.7
Fat-free mass (kg)	41.9 ± 4.7	42.4 ± 5.0

*Values are means ± sd. n, number of participants. There were no significant differences between groups*.

**Figure 2:**
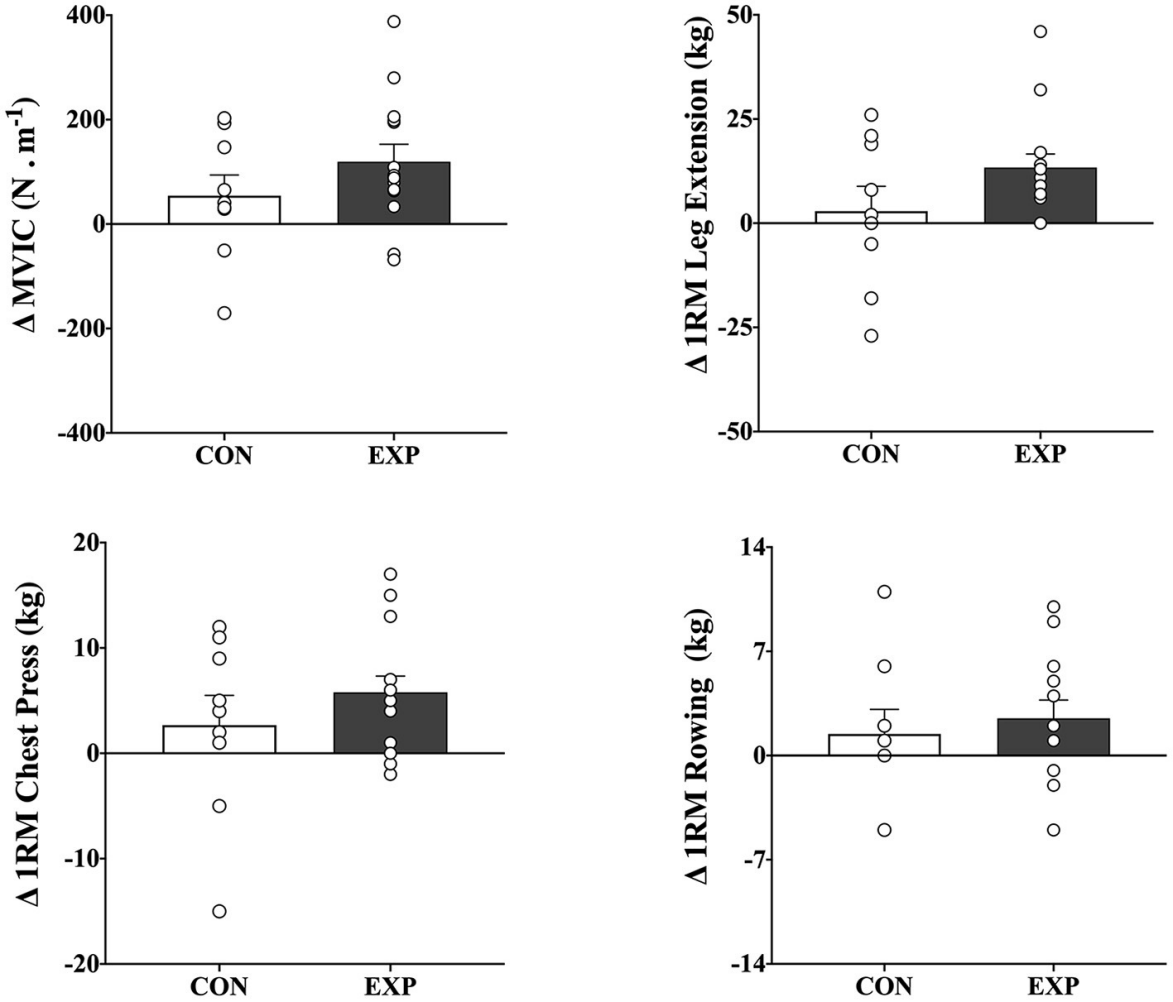
Mean and individual change scores in maximal voluntary isometric contraction (mvic; *top left*), 1rm leg extension (*top right*), 1rm chest press (*bottom left*), and 1rm seated row (*bottom right*) after combined endurance and resistance training for both control (con; *n* = 9) and experimental (exp; *n* = 9) groups. The values are post- minus pretraining (means ±sem). There were no significant differences between groups.

Table 2 shows baseline and change scores for each group, as well as the *P* values for the two-tailed, paired *t*-tests that indicate which within-group change scores were significant. There were significant increases in mvic (119.5 N.m^−1^, 95% ci 47.8–191.2), 1rm leg extension (13.3 kg, 95% ci 6.3–20.4), and 1rm chest press (5.7 kg, 95% ci 2.4–9.1) following the intervention period in the exp group (*p* <0.05), but not in the con group (*p* > 0.05). There were, however, no significant between-group differences in mvic and 1rm chest press, despite a trend toward significance for 1rm leg extension [*f* (1, 23) = 2.655, *p* = 0.119] (Figure 2). There were also no significant within- or between-group differences in 1rm seated row (see Table 2, Figure 2 for details). Of note, there were no significant baseline differences between groups for all variables (*p* > 0.05).

**Table 2:** Baseline values, changes scores, and significance of change scores in both CON and EXP groups.

**Variable**	**EXP** **group (** ***n*** **=** **14)** **^a^**			**CON** **group (** ***n*** **=** **9)**		
	**Baseline**	**Change**	***P* value**	**Baseline**	**Change**	***P* value**
Training						
MVIC (N.m^−1^)	461.0 ± 284.9	119.5 ± 124.2	<0.01	535.9 ± 302.9	54.2 ± 118.8	0.20
1RM leg extension (kg)	52.6 ± 18.6	13.3 ± 12.2	<0.01	54.7 ± 28.0	2.8 ± 17.8	0.64
1RM chest press (kg)	30.4 ± 10.4	5.7 ± 5.7	<0.01	27.7 ± 12.3	2.6 ± 8.5	0.37
1RM seated row (kg)	37.2 ± 9.7	2.5 ± 4.6	0.06	34.3 ± 14.7	1.4 ± 4.9	0.40
Detraining						
MVIC (N.m^−1^)	573.6 ± 293.7	33.6 ± 128.8	0.36			
1RM leg extension (kg)	65.8 ± 26.5	−6.3 ± 12.4	0.08			
1RM chest press (kg)	36.9 ± 10.0	−2.4 ± 7.2	0.24			
1RM seated row (kg)	39.4 ± 10.3	−2.0 ± 3.9	0.09			

*Values are means ± SD. n, number of participants*.

*MVIC, maximal voluntary isometric contraction. 1RM, one repetition maximum*.

a *There were no significant baseline differences between groups. The values per detraining refer to participants who have both baseline and detraining data, and, therefore, the number of participants in detraining (n = 13) is lower than in training (n = 14)*.

**Figure 3:**
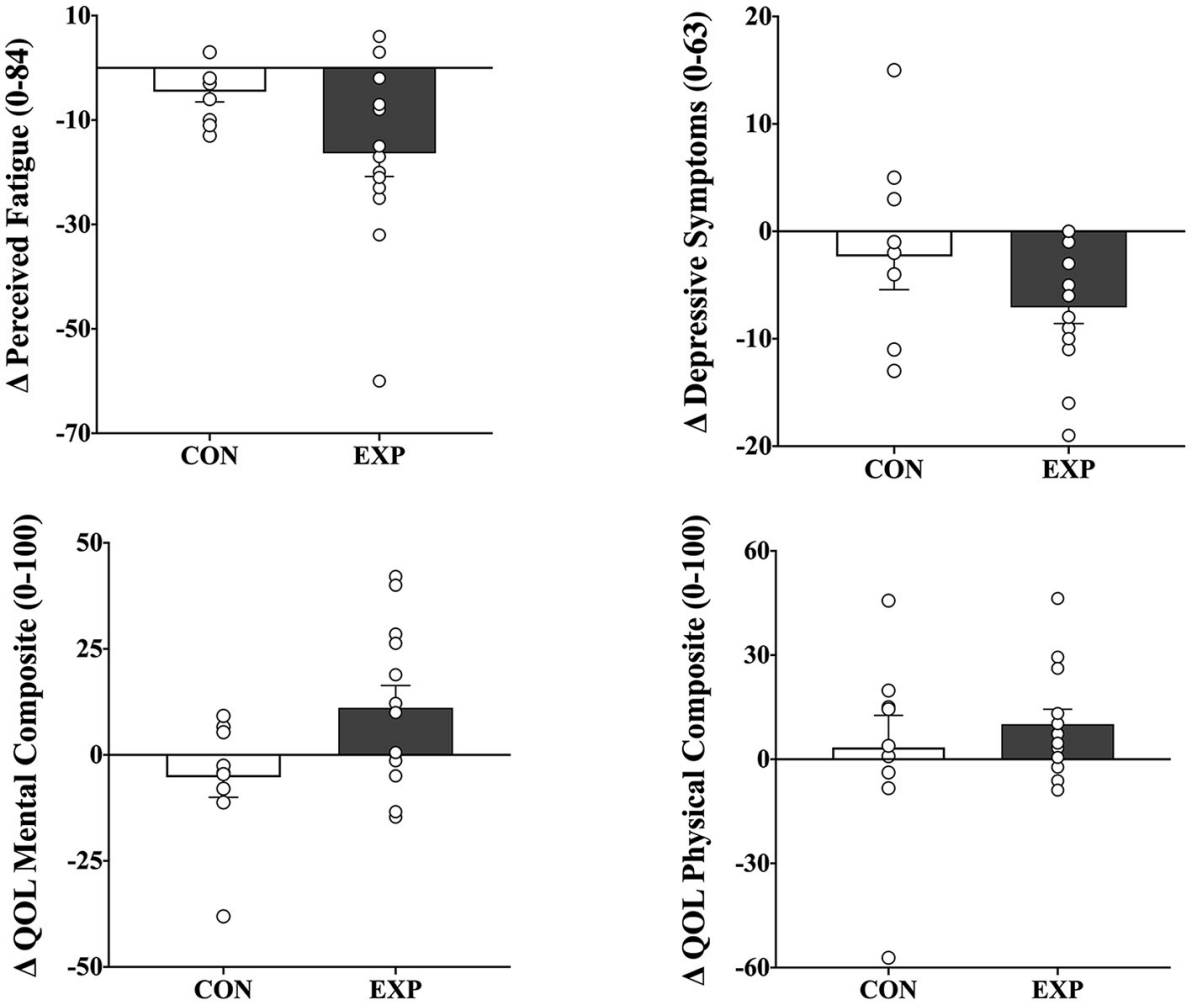
Mean and individual change scores in perceived fatigue (*top left*), depressive symptoms (*top right*), and both mental-(*bottom left*) and physical-(*bottom right*) composites of health-related quality of life after combined endurance and resistance training for both control (con; *n* = 9) and experimental (exp; *n* = 9) groups. The values are post- minus pretraining (means ±sem). There were no significant differences between groups.

Table 3 shows baseline and change scores for each group, as well as the *p* values for the two-tailed, paired *t*-tests that indicate which within-group change scores were significant. There were significant reductions in perceived fatigue (−16.3 units, 95% ci −25.9–−6.7) and depressive symptoms (−7.0 units, 95% ci −10.3–−3.8) and improvements in both physical- (10.0 units, 95% ci.6 – 19.5) and mental (11.1 units, 95% ci −0.3–22.5) composites of the health-related quality of life following the intervention period in the exp group (*p* <0.05), but not in the con group (*p* > 0.05). There were also between-group differences in fatigue [*f* (1, 23) = 6.103, *p* = 0.023] and the mental composite of quality of life [*f* (1, 23) = 5.660, *p* = 0.028]. There were, however, no significant between-group differences in depression and the physical composite of the quality of life (*p* > 0.05) (Figure 3). There were also no significant baseline differences between groups for all variables (*p* > 0.05).

**Table 3:** Baseline values, changes scores, and significance of change scores in both CON and EXP groups.

**Variable**	**EXP** **group (** ***n*** **=** **14)** **^a^**			**CON** **group (** ***n*** **=** **9)**		
	**Baseline**	**Change**	***P* value**	**Baseline**	**Change**	***p* value**
Training						
Perceived fatigue (0–84)	39.9 ± 15.0	−16.3 ± 16.6	<0.01	44.8 ± 16.3	−4.5 ± 5.8	0.47
Depressive symptoms (0–63)	16.6 ± 9.3	−7.0 ± 5.6	<0.01	15.4 ± 7.2	−2.3 ± 9.2	0.05
QOL mental composite (0–100)	48.6 ± 19.3	11.1 ± 18.9	<0.05	51.5 ± 18.2	−5.2 ± 14.1	0.29
QOL physical composite (0–100)	57.5 ± 22.4	10.0 ± 15.5	<0.05	55.4 ± 23.8	3.3 ± 27.7	0.72
Detraining						
Perceived fatigue (0–84)	23.7 ± 16.4	9.2 ± 11.0	<0.05			
Depressive symptoms (0–63)	10.3 ± 7.7	−0.6 ± 5.3	0.64			
QOL mental composite (0–100)	59.7 ± 14.6	−6.5 ± 18.0	0.21			
QOL physical composite (0–100)	67.6 ± 17.7	−5.1 ± 13.9	0.20			

*Values are means ± SD. n, number of participants*.

*QOL, health-related quality of life*.

a *There were no significant baseline differences between groups. The values per detraining refer to participants who have both baseline and detraining data, and, therefore, the number of participants in detraining (n = 13) is lower than in training (n = 14)*.

The residual effects of the combined exercise training were also investigated following a 12-wk period of training cessation, and thus all participants in the exp group were asked to discontinue exercise training for 12 weeks after their last training session. Two-tailed, paired *t*-tests indicated no significant changes for any of the measures of muscle strength, depression, and quality of life following the detraining period (see Tables 2, 3 for details), except for fatigue.

Test-retest reliability for all studied variables was examined for the preintervention vs. postintervention measurements from the con group. The icc values for measures of muscle strength (0.95, 0.81, 0.86, and 0.96, respectively for mvic, 1rm leg extension, 1rm chest press, and 1rm chest press), fatigue (0.96), and the physical composite of the quality of life (0.83), were relatively high. The icc values for both depression (0.57) and the mental composite of the quality of life (0.52), otherwise, were relatively low.

## Discussion

In recent years, endurance training has emerged as an effective and well-tolerated non-pharmacologic intervention for persons with ms (16, 20). It has been speculated, however, that endurance training, combined with resistance training, may further augment the adaptations to exercise training in this population (20). Unfortunately, to the best of our knowledge, there is scarce information on the effects of combined exercise training in people with ms (19, 21, 22). The present study, therefore, examined the effectiveness of a 12-wk combined endurance and resistance training program on muscle strength, perceived fatigue, depressive symptoms, and health-related quality of life in women with ms. The residual effects of this combined training regimen were also investigated following a 12-wk period of training cessation. The major finding was that the combination of endurance and resistance training caused significant increases in mvic, 1rm leg extension, and 1rm chest press. These improvements in muscle strength following the intervention period occurred in tandem with beneficial effects on measures of fatigue, depression, and quality of life. These changes, however, were only significant for the exp group and persisted after 12 weeks of detraining (see below for details). The results of the present study, therefore, have potentially important clinical implications and suggest that combined exercise training, at a minimum, may prevent the disease-related deterioration of muscular performance and quality of life and well-being in persons with ms.

Previous research has reported that most people with ms may experience reduced muscle strength during dynamic and isometric muscle contractions (5). The reasons for the strength impairments in this population are still unclear but may be related to mechanisms of both neural and muscular origin (6). Whether ms-related decreased muscle strength occurs due to the disease, muscle disuse, or both is also uncertain (9), but many researchers believe that detrimental changes in muscle strength may contribute to functional limitations and mobility impairments in people with ms (15, 39). Some reports suggest that resistance training, alone or combined with endurance training, can be beneficial in maintaining or improving muscle strength (12–14), with potential clinical implications for persons with ms. The current results support this notion and suggest that a 12-wk combined endurance and resistance training program promoted changes in muscle strength. These significant changes represented average muscle strength changes of ~27% (sd 29.0), ~23% (sd 26.8), and ~40% (sd 48.8), respectively for mvic, 1rm leg extension, and 1rm chest press. Despite the large variability, the magnitude of these changes was as great as the changes observed in an earlier work (15), where significant increases of ~15% for mvic and ~37% 1rm leg press after a progressive resistance training regimen were reported. The findings of increases in muscle strength following combined exercise training, along with those from other recent investigations (12–15, 38), may have important implications in ms rehabilitation for counteracting the well-documented disease-related manifestation of muscle weakness in persons with ms.

The improvements in muscle strength persisted after 12 weeks of detraining. It is essential to point out, however, that these positive changes following both training and detraining were only significant for the exp group, and no significant differences in change scores among the groups were reported. Some possible explanations exist. It is possible that with fewer participants in the con group (*n* = 9, compared with *n* = 14 for the exp group), there was decreased power to detect significant between-group differences in muscle strength. Alternatively, in most cases, there were noticeable variations in the change scores (as indicated by the sd of the changes; see Table 2 for details), which would indicate that the large data variability somewhat mitigated both training and detraining effects. The reasons for this large variability are unclear, but clinical manifestations of ms broadly vary between individuals, based on the extent and location of the lesions and disease exacerbations and progression (4). Both hypotheses, however, are naturally speculative and await further investigation. Another explanation could be that the training stimulus was not high enough to promote favorable changes in muscle strength in persons with ms. Whether the endurance training mitigated the gains in muscular performance promoted by the resistance training – or vice-versa - is also uncertain. These hypotheses, however, are plausible since the icc values for measures of muscle strength were relatively high and, hence, are not due to poor test-retest reliability (38).

Another surprising finding is that no correlations were found between training-induced changes in muscle strength and perceived fatigue (unpublished data; range *r* = −0.21 to 0.26, *p* > 0.05). Muscle weakness and fatigue are the most common ms-related manifestations and occur at all stages of the disease. The prevalence of fatigue in people with ms is elevated, occurring at a rate as high as 55% (40). The neurobiology of fatigue in ms, however, is still not well understood but appears to be related to pathological processes underlying ms, with a significant contribution of multiple psychological factors (41). It is clear, otherwise, that fatigue is a multidimensional construct that influences both physical and mental ability and effort. Previous research has indicated that resistance (12–14, 42) and endurance training (16) can positively impact both physical and mental manifestations of fatigue in persons with ms. The results of the present study are in line with these reports and confirm attenuated symptoms of fatigue after combined exercise training. Notably, eight participants defined as fatigued at baseline (mfis score above 36) reported discrete fatigue symptoms following the intervention period. These beneficial effects in terms of fatigue, however, were not sustained over time.

Psychological depression is an important public health problem and is considered one of the leading causes of disability worldwide. The prevalence of depression in persons with ms is high, occurring at a rate ~5 times that of the general population (43). Because of its high prevalence, importance to the quality of life and well-being (43), and impact on the disease course itself (44), depression has been intensively studied in ms. Unfortunately, there are scarce information on the usefulness of resistance training, alone or combined with endurance training, to prevent depression in ms. Some reports have indicated that both resistance (23, 42) and endurance training (17, 18) can impact psychological depression in persons with ms. The results of the present study are in line with these findings and confirm that a significant reduction in depressive symptoms after combined exercise training was evident. Notably, four participants at considerable risk for depression at baseline (bdi-ii score of 19 or above) reported discrete symptoms of depression following the intervention period. Similar findings were observed in health-related quality of life, where improvements in physical and mental composites of quality of life were found. These results are consistent with previous reports (12, 17, 18, 42) and are essential because the quality of life is often reduced in persons with ms (45). Health-related quality of life is also important because it reflects how a person feels in the physical, mental, and social aspects of life after an intervention such as exercise (18). It is largely unknown whether the protective effects of a combined exercise regimen on measures of depression and quality of life are sustained over time, but most effects persisted even after cessation of training.

Some strengths and limitations within the present study warrant mention. The most prominent strength was the nature of the combination program, permitting to describe the synergistic effects (and their residual effects) of both endurance and resistance training. The randomized controlled trial design is another strength of this study since few ms-related studies have embraced this methodological approach (20, 42, 46). The sample is, otherwise, the primary limitation of this study. The current sample is relatively narrow in terms of disease status, with most participants defined as mildly disabled “patients” (mean edss score 2.25, sd 0.8; see Supplementary Table for details). It is, therefore, difficult to generalize our findings to people with advanced ms and progressive clinical courses (11). The small sample size is another limitation of the current study. The reasons for the high dropout (*n* = 3 of 13 volunteers) in the con group are uncertain, but previous work (15, 42) suggests that participants in the “waiting list” may feel they are deselected at the expense of the participants in the experimental group. The current findings should, therefore, be interpreted with caution until they have been confirmed by adequately powered follow-up studies. Lastly, a variety of common fluid and serum markers of clinical importance in people with ms were not assessed in the current study. Future studies are therefore necessary to further elucidate the impact of both endurance and resistance training on molecular markers in this population.

This study provides the first description of the effectiveness of implementing a combined endurance and resistance training program for persons with ms. The current results suggest that this exercise training regimen results in increases in measures of muscle strength, which are accompanied by improvements in perceived fatigue, depressive symptoms, and health-related quality of life. These changes persisted even after 12 weeks of detraining. The findings of the present study, therefore, have potentially important clinical implications for the management of ms and encourage exercise professionals, physicians, and other healthcare providers to support combined exercise training as a non-pharmacologic approach capable of preventing the disease-related deterioration of muscular performance and quality of life and well-being.

## Data Availability Statement

The raw data supporting the conclusions of this article will be made available by the authors, without undue reservation.

## Ethics Statement

The studies involving human participants were reviewed and approved by IRB Fondazione IRCSS San Matteo. The patients/participants provided their written informed consent to participate in this study.

## Author Contributions

LC, CFB, MV, CM, and RB: Conceived and designed the study. LC, GL, EC, and GM: Performed the study. LC, CFB, and CM: Analysed the data. LC and CFB: Wrote the manuscript. LC, CFB, GL, EC, GM, MV, CM, and RB: Reviewed the manuscript. All authors contributed to the article and approved the submitted version.

## Conflict of Interest

The authors declare that the research was conducted in the absence of any commercial or financial relationships that could be construed as a potential conflict of interest.

## Publisher's Note

All claims expressed in this article are solely those of the authors and do not necessarily represent those of their affiliated organizations, or those of the publisher, the editors and the reviewers. Any product that may be evaluated in this article, or claim that may be made by its manufacturer, is not guaranteed or endorsed by the publisher.

## References

[1] Confavreux C Vukusic S Adeleine P. Early clinical predictors and progression of irreversible disability in multiple sclerosis: an amnesic process. Brain. (2003) 126:770–82. 10.1093/brain/awg081 12615637

[2] Mayr WT Pittock SJ McClelland RL Jorgensen NW Noseworthy JH Rodriguez M. Incidence and prevalence of multiple sclerosis in Olmsted County, Minnesota, 1985-2000. Neurology. (2003) 61:1373–7. 10.1212/01.wnl.0000094316.90240.eb 14638958

[3] GBD 2016 Neurology Collaborators. Global, regional, and national burden of neurological disorders, 1990-2016: a systematic analysis for the GBD study 2016. Lancet Neurol. (2019) 18:459–80. 10.1016/S1474-4422(18)30499-X 30879893 PMC6459001

[4] Crayton HJ Rossman HS. Managing the symptoms of multiple sclerosis: a multimodal approach. Clin Ther. (2006) 28:445–60. 10.1016/j.clinthera.2006.04.005 16750459

[5] Jørgensen M Dalgas U Wens I Hvid LG. Muscle strength and power in persons with multiple sclerosis: a systematic review and meta-analysis. J Neurol Sci. (2017) 376:225–41. 10.1016/j.jns.2017.03.022 28431618

[6] Ng AV Miller RG Gelinas D Kent-Braun JA. Functional relationships of central and peripheral muscle alterations in multiple sclerosis. Muscle Nerve. (2004) 29:843–52. 10.1002/mus.20038 15170617

[7] Rice CL Vollmer TL Bigland-Ritchie B. Neuromuscular responses of patients with multiple sclerosis. Muscle Nerve. (1992) 15:1123–32. 10.1002/mus.880151011 1406770

[8] Van der Kamp W Maertens de. Noordhout A, Thompson PD, Rothwell JC, Day BL, Marsden CD. Correlation of phasic muscle strength and corticomotoneuron conduction time in multiple sclerosis. Ann Neurol. (1991) 29:6–12. 10.1002/ana.410290104 1996880

[9] Kent-Braun JA Sharma KR Weiner MW Miller RG. Effects of exercise on muscle activation and metabolism in multiple sclerosis. Muscle Nerve. (1994) 17:1162–9. 10.1002/mus.880171006 7935523

[10] Garner DJ Widrick JJ. Cross-bridge mechanisms of muscle weakness in multiple sclerosis. Muscle Nerve. (2003) 27:456–64. 10.1002/mus.10346 12661047

[11] Trapp BD Nave KA. Multiple sclerosis: an immune or neurodegenerative disorder? Annu Rev Neurosci. (2008) 31:247–69. 10.1146/annurev.neuro.30.051606.094313 18558855

[12] Dodd KJ Taylor NF Shields N Prasad D McDonald E Gillon A. Progressive resistance training did not improve walking but can improve muscle performance, quality of life and fatigue in adults with multiple sclerosis: a randomized controlled trial. Mult Scler. (2011) 17:1362–74. 10.1177/1352458511409084 21677021

[13] Hayes HA Gappmaier E LaStayo PC. Effects of high-intensity resistance training on strength, mobility, balance, and fatigue in individuals with multiple sclerosis: a randomized controlled trial. J Neurol Phys Ther. (2011) 35:2–10. 10.1097/NPT.0b013e31820b5a9d 21475078

[14] White LJ McCoy S Castellano V Gutierrez G Stevens JE Walter GA. Resistance training improves strength and functional capacity in persons with multiple sclerosis. Mult Scler. (2004) 10:668–74. 10.1191/1352458504ms1088oa 15584492

[15] Dalgas U Stenager E Jakobsen J Petersen T Hansen HJ Knudsen C. Resistance training improves muscle strength and functional capacity in multiple sclerosis. Neurology. (2009) 73:1478–84. 10.1212/WNL.0b013e3181bf98b4 19884575

[16] Mostert S Kesselring J. Effects of a short-term exercise training program on aerobic fitness, fatigue, health perception and activity level of subjects with multiple sclerosis. Mult Scler. (2002) 8:161–8. 10.1191/1352458502ms779oa 11990874

[17] Petajan JH Gappmaier E White AT Spencer MK Mino L Hicks RW. Impact of aerobic training on fitness and quality of life in multiple sclerosis. Ann Neurol. (1996) 39:432–41. 10.1002/ana.410390405 8619521

[18] Sutherland G Andersen MB Stoove MA. Can aerobic exercise training affect health-related quality of life for people with multiple sclerosis? J Sport Exerc Psych. (2007) 23:122–35. 10.1123/jsep.23.2.12229791509

[19] Romberg A Virtanen A Ruutiainen J Aunola S. Karppi, SL, Vaara M, et al. Effects of a 6-month exercise program on patients with multiple sclerosis: a randomized study. Neurology. (2004) 63:2034–8. 10.1212/01.wnl.0000145761.38400.65 15596746

[20] Dalgas U Stenager E Ingemann-Hansen T. Multiple sclerosis and physical exercise: recommendations for the application of resistance-, endurance- and combined training. Mult Scler. (2008) 14:35–53. 10.1177/1352458507079445 17881393

[21] Romberg A Virtanen A Ruutiainen J. Long-term exercise improves functional impairment but not quality of life in multiple sclerosis. J Neurol. (2005) 252:839–45. 10.1007/s00415-005-0759-2 15765197

[22] Grazioli E Tranchita E Borriello G Cerulli C Minganti C Parisi A. The effects of concurrent resistance and aerobic exercise training on functional status in patients with multiple sclerosis. Curr Sports Med Rep. (2019) 18:452–7. 10.1249/JSR.0000000000000661 31834177

[23] Dalgas U Stenager E Sloth M Stenager E. The effect of exercise on depressive symptoms in multiple sclerosis based on a meta-analysis and critical review of the literature. Eur J Neurol. (2015) 22:443–e34. 10.1111/ene.12576 25327395

[24] Polman CH Reingold SC Banwell B Clanet M Cohen JA Filippi M. Diagnostic criteria for multiple sclerosis: 2010 revisions to the McDonald criteria. Ann Neurol. (2011) 69:292–302. 10.1002/ana.22366 21387374 PMC3084507

[25] Kurtzke JF. Rating neurologic impairment in multiple sclerosis: an expanded disability status scale (edss). Neurology. (1983) 33:1444–52. 10.1212/wnl.33.11.1444 6685237

[26] American College of Sports Medicine. Guidelines for Exercise Testing and Prescription. Philadelphia: Lippincott Williams & Wilkins (2014).

[27] De Souza-Teixeira F Costilla S Ayán C García-López D González-Gallego J de Paz JA. Effects of resistance training in multiple sclerosis. Int J Sports Med. (2009) 30:245–50. 10.1055/s-0028-1105944 19199197

[28] Karpatkin HI Cohen ET Klein S Park D Wright C Zervas M. The effect of maximal strength training on strength, walking, and balance in people with multiple sclerosis: a pilot study. Mult Scler Int. (2016) 2016:5235971. 10.1155/2016/5235971 28116161 PMC5220488

[29] Kos D Kerckhofs E Carrea I Verza R Ramos M Jansa J. Evaluation of the Modified Fatigue Impact Scale in four different European countries. Mult Scler. (2005) 11:76–80. 10.1191/1352458505ms1117oa 15732270

[30] Fisk JD Ritvo PG Ross L Haase DA Marrie TJ Schlech WF. Measuring the functional impact of fatigue: initial validation of the Fatigue Impact Scale. Clin Infect Dis. (1994) 18:S79–83. 10.1093/clinids/18.supplement_1.s79 8148458

[31] Flachenecker P Kumpfel T Kallmann B Gottschalk M Grauer O Rieckmannet P. Fatigue in multiple sclerosis: a comparison of different rating scales and correlation to clinical parameters. Mult Scler. (2002) 8:523–6. 10.1191/1352458502ms839oa 12474995

[32] Ghisi M Flebus GB Montano A. Beck Depression Inventory – II (bdi-ii) Manuale. Firenze: OS Organizzazioni Speciali. (2006).

[33] Beck AT Steer RA Brown GA. Beck Depression Inventory: Second Edition Manual. San Antonio: the Psychological Corporation. (1996).

[34] Solari A Filippini G Mendozzi L Ghezzi A Cifani S Barbieri E. Validation of Italian multiple sclerosis quality of life 54 questionnaire. J Neurol Neurosurg Psychiatry. (1999) 67:158–62. 10.1136/jnnp.67.2.158 10406981 PMC1736469

[35] Vickrey BG Hays RD Harooni R Myers LW Ellison GW A. health-related quality of life measure for multiple sclerosis. Qual Life Res. (1995) 4:187–206. 10.1007/BF02260859 7613530

[36] Taheri M Negahban H Mostafaee N Salehi R Tabesh H. Responsiveness of selected outcome measures of participation restriction and quality of life in patients with multiple sclerosis. Disabil Rehabil. (2016) 38:482–6. 10.3109/09638288.2015.1044622 25955822

[37] Vickers AJ Altman D. Statistics notes: analysing controlled trials with baseline and follow-up measurements. BMJ. (2001) 323:1123–4. 10.1136/bmj.323.7321.1123 11701584 PMC1121605

[38] Weir JP. Quantifying test-retest reliability using the intraclass correlation coefficient and the SEM. J Strength Cond Res. (2005) 19:231–40. 10.1519/15184.1 15705040

[39] Thoumie P Mevellec E. Relation between walking speed and muscle strength is affected by somatosensory loss in multiple sclerosis. J Neurol Neurosurg Psychiatry. (2002) 73:313–5. 10.1136/jnnp.73.3.313 12185167 PMC1738010

[40] Fisk JD Pontefract A Ritvo PG Archibald CJ Murray TJ. The impact of fatigue on patients with multiple sclerosis. Can J Neurol Sci. (1994) 21:9–14. 8180914

[41] Veldhuijzen van Zanten J Douglas MR Ntoumanis N. Fatigue and fluctuations in physical and psychological wellbeing in people with multiple sclerosis: a longitudinal study. Mult Scler Relat Disord. (202) 47:102602. 10.1016/j.msard.2020.102602 33176231

[42] Dalgas U Stenager E Jakobsen J Petersen T Hansen HJ Knudson C. Fatigue, mood and quality of life improve in ms patients after progressive resistance training. Mult Scler. (2010) 16:480–90. 10.1177/1352458509360040 20194584

[43] Arnett PA Barwick FH Beeney JE. Depression in multiple sclerosis: review and theoretical proposal. J Int Neuropsychol Soc. (2008) 14:691–724. 10.1017/S1355617708081174 18764967

[44] Miller A Dishon S. Health-related quality of life in multiple sclerosis: the impact of disability, gender and employment status. Qual Life Res. (2006) 15:259–71. 10.1007/s11136-005-0891-6 16468081

[45] Mohr DC Goodkin DE Bacchetti P Boudewyn AC Huang L Marrietta P. Psychological stress and the subsequent appearance of new brain mri lesions in ms. Neurology. (2000) 55:55–61. 10.1212/wnl.55.1.55 10891906

[46] Motl R Pilutti L. The benefits of exercise training in multiple sclerosis. Nat Rev Neurol. (2012) 8:487–97. 10.1038/nrneurol.2012.136 22825702

