# Supplementary material for: Effects of Combined Endurance and Resistance Training in Women With Multiple Sclerosis: A Randomized Controlled Study
Source: Front Neurol. 2021 Aug 5;12:698460. doi: 10.3389/fneur.2021.698460 (PMC8374042; doi:10.3389/fneur.2021.698460)
Supplement: Supplementary file 1 [file Data_Sheet_1.PDF]

Supplementary Table 1

Table 1. Additional characteristics of the participants at baseline.

Age (yr)	46.0	±	7.2
Mean disease duration (yr)	14.6	±	6.9
Expanded Disability Status Scale (EDSS)	2.25	±	0.8
Treatments			
No treatment		5	(21.7%)
Interferons		6	(26.1%)
Dimethyl fumarate		5	(21.7%)
Teriflunomide		3	(13.0%)
Fingolimod		2	(8.7%)
Natalizumab		2	(8.7%)

Values are means ± SD or frequency (percentage).
